# A Case Of Awake Percutaneous Extracorporeal Membrane Oxygenation For High-risk Percutaneous Coronary Intervention

**DOI:** 10.7759/cureus.1191

**Published:** 2017-04-25

**Authors:** Shuangbo Liu, Amir Ravandi, Malek Kass, Basem Elbarouni

**Affiliations:** 1 Section of Cardiology, St. Boniface Hospital, University of Manitoba, Canada

**Keywords:** ecmo, pci, venoarterial extracorporeal membrane oxygenation (va-ecmo), percutaneous coronary intervention

## Abstract

With significant improvements in percutaneous coronary intervention (PCI) technology, complex high risk PCI is increasingly offered to patients with limited revascularization options. Percutaneous mechanical circulatory support devices are often utilized for hemodynamic support during these complex procedures. Veno-arterial extracorporeal membrane oxygenation (ECMO) allows full hemodynamic support and provides systemic oxygenation. We describe a case of left main bifurcation stenting performed at our center with ECMO support in an awake patient without general anesthesia.

## Introduction

Significant improvements in percutaneous coronary intervention (PCI) technology has led to complex high-risk PCI being offered to patients with limited revascularization options. Percutaneous mechanical circulatory support (MCS) devices, including veno-arterial extracorporeal membrane oxygenation (VA-ECMO), are often utilized for hemodynamic support during these complex procedures. VA-ECMO provides cardiopulmonary support and is comprised of a venous cannula to drain the deoxygenated blood, centrifugal nonpulsatile pump, membrane for gas exchange and an arterial cannula to return the oxygenated blood to the body [1]. This allows for full hemodynamic support, with cardiac outputs over 4.5 L/min [2].

The definition of high-risk PCI is currently evolving. Patient, lesion and clinical presentation risk factors need to be considered when making the decision about need for percutaneous hemodynamic support [1]. Commonly considered factors include increased age, reduced left ventricular systolic function, ACC/AHA Type C lesions, vessels that supply a large area (unprotected left main, last patent vessel or severe 3 vessel disease) or acutely ill patients (large myocardial infarction or cardiogenic shock) [1]. No randomized clinical trial exists to assess the use of ECMO in high-risk PCI.

## Case presentation

**Figure 1:**
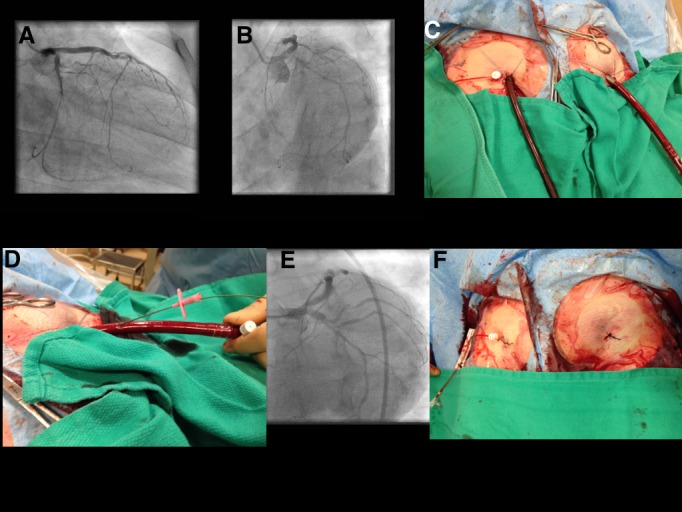
Case illustration Figure 1A: Pre-procedure coronary angiography (AP caudal projection) demonstrating severe distal left main stenosis involving left main bifurcation Figure 1B: Pre-procedure coronary angiography (left anterior oblique caudal projection) demonstrating severe distal left main stenosis involving left main bifurcation Figure 1C: Intra-procedure image demonstrating arterial and venous cannulation Figure 1D: Fluoroscopy image (left anterior oblique caudal projection) demonstrating the left coronary system after successful PCI Figure 1E: End of procedure image demonstrating arterial cannula puncture and wiring to maintain vascular access Figure 1F: After successful ECMO decannulation and hemostasis

A 54-year old male with a history of ischemic heart disease and hypertension presented with an acute coronary syndrome (ACS). Coronary angiography demonstrated a left dominant system with a 90% stenosis in the distal left main (LM) extending into the left circumflex (Cx) and left anterior descending (LAD) arteries, in addition to a previously documented 100% mid-LAD occlusion (Figure 1A, 1B). Left ventricular (LV) ejection fraction was estimated at 30-40% by transthoracic echocardiogram. He was not a surgical candidate given the lack of graftable targets. The patient expressed wished to pursue revascularization due to ongoing symptoms. We anticipated that wiring the Cx would be challenging due to an acute takeoff angle with a high risk of acute vessel closure. Given the reduced LV systolic function as well as complex anatomy, we discussed the need for percutaneous MCS. In discussion with our Heart Team, we proceeded with high-risk PCI with awake ECMO support.

The patient was lightly sedated with midazolam and fentanyl. PCI was performed via the right femoral artery using a 7F sheath. ECMO cannulation (Figure 1C) was via the left femoral artery (17F) and right femoral vein (24/29F 2-stage venous cannula). We pre-closed both access sites with two Perclose ProGlide (Abbott Vascular, Redwood City, CA) placed at orthogonal angles of 10 and 2 o’clock. ECMO flow was set at 3.5 L/min with 3360 RPM using a BioMedicus 540 centrifugal pump (Medtronic, Minneapolis, MN). A SuperCross micro-catheter with a preformed 1200 angled tip (Vascular Solutions, Minneapolis, MN) was used and the Cx was wired using a Sion wire (ASAHI, Abbott vascular, Redwood City, CA). PCI was performed with excellent results using a T-and protrusion (TAP) bifurcation technique with two drug-eluting stents (Figure 1D). The patient was hemodynamically stable throughout the procedure with no chest pain despite prolonged and high-pressure inflations in the LM. After PCI, the patient was weaned off ECMO and decannulated successfully (Figure 1E) in the cath lab. To maintain vascular access, we punctured and passed a 260 cm J wire through both ECMO cannulas before removing them (Figure 1F). A third Perclose at 12 o’clock was needed to achieve hemostasis in both access sites. The patient was transferred to the ward, discharged two days later, and was doing well at one month follow up.

 

## Discussion

With significant improvements in PCI technology, complex high-risk PCI is increasingly offered to patients with limited revascularization options. Percutaneous MCS devices are often utilized for hemodynamic support during these complex procedures.

IABP is the most common type of MCS used during PCI, mainly due to its ease of use. A single double lumen catheter with a balloon at the end is inserted and attached to a pump. Helium inflates the balloon at the beginning of diastole. IABP increases diastolic blood pressure and coronary artery perfusion decreases afterload and myocardial oxygen consumption and may increase cardiac output slightly [1]. Advantages include being available in most centers and ease of use. A disadvantage of IABP is the need for a stable cardiac rhythm and not being able to provide full hemodynamic support.

Another type of MCS is the Impella (Abiomed, Danvers, Massachusetts), which is a device placed across the aortic valve. When optimally positioned, it propels blood from the LV to the aorta. The Impella decreases end-diastolic wall stress while increasing diastolic compliance as well as aortic and intracoronary pressures and coronary flow velocity reserve [1].  Advantages include enhanced cardiac output (2.5-4 L/min, depending on cannula size) and does not require a stable cardiac rhythm to function. However, it may not be universally available, requires larger cannula size and therefore higher risk of complications compared to IABP.

While traditionally, IABP and Impella are used, the former might not provide adequate hemodynamic support [1] and the latter can be quite expensive. VA-ECMO allows full hemodynamic support and provides systemic oxygenation [2]. Other advantages include ease of use, the ability to augment cardiac output by >3.5 L/minute and unlike IABP, does not require a stable cardiac rhythm for optimal function [1]. It also provides better circulatory support at a reduced cost in comparison to Impella. Limitations include local expertise, the need for a larger arterial cannula, and the need to cannulate the femoral vein. Post-procedural care of ECMO patients can be challenging and resource intensive, but as demonstrated in our case, avoiding intubation and general anesthesia by performing awake ECMO with successful percutaneous closure and hemostasis allows for rapid mobilization and patient discharge.  This is the second case of awake ECMO performed at our center; both with a favorable outcome and successful percutaneous hemostasis [3]. Both cases demonstrate the safety and feasibility of awake ECMO in high-risk PCI procedures. 

## Conclusions

Percutaneous mechanical circulatory support devices, including awake VA-ECMO, can be safely performed in appropriate cases of high-risk PCI. 
